# Acetonitrile­{3-[bis­(2-pyridyl­methyl-κ*N*)amino-κ*N*]propanol-κ*O*}(perchlorato-κ*O*)copper(II) perchlorate

**DOI:** 10.1107/S1600536810053985

**Published:** 2011-01-08

**Authors:** Jong Won Shin, Sankara Rao Rowthu, Hyun Jung Cho, Kil Sik Min

**Affiliations:** a Department of Chemistry, Kyungpook National University, Daegu 702-701, Republic of Korea; b Department of Chemistry Education, Kyungpook National University, Daegu 702-701, Republic of Korea

## Abstract

In the title compound, [Cu(ClO_4_)(C_2_H_3_N)(C_15_H_19_N_3_O)]ClO_4_, the Cu^II^ ion is coordinated by three N atoms and a hydroxyl-O atom of the tetra­dentate ligand, an O atom of a perchlorate ion and an N atom of an acetonitrile ligand giving a tetra­gonally distorted octa­hedral environment around the copper(II) atom. There is an offset inter-complex face-to-face π–π inter­action [centroid–centroid distance = 3.718 (2) Å] involving one of the pyridine rings of the ligand as well as an intra-complex O—H⋯O hydrogen-bonding inter­action between the coordinated hydroxyl group of the ligand and the perchlorate counter-ion.

## Related literature

The preparation and characterization of polyamine complexes have allowed the elucidatation of the mechanisms of metalloenzyme reactions, see: Tshuva & Lippard (2004 ▶). For studies of complexes with bis­(2-pyridyl­meth­yl)amine moieties, see: Bebout *et al.* (1998 ▶); Shin *et al.* (2010 ▶). For potential biological applications of the tridentate unit, see: van Staveren *et al.* (2002 ▶). Examples include the use of Pd^II^ and Pt^II^ complexes with bis­(2-pyridyl­meth­yl)amine or its derivatives as anti­cancer agents, e.g. *cis*-platin (Rauterkus *et al.*, 2003 ▶). For inter­complex π–π stacking inter­actions, see: Shetty *et al.* (1996 ▶). For the preparation of *N*,*N*-bis­(2-pyridyl­meth­yl)-3-amino­propanol, see: Young *et al.* (1995 ▶).
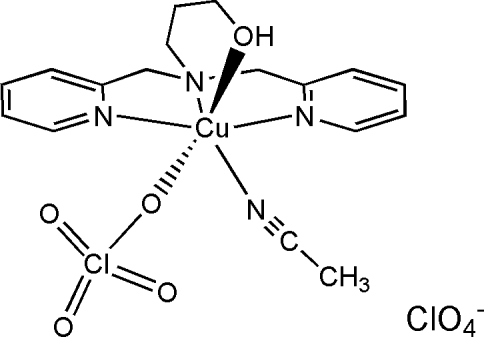


## Experimental

### 

#### Crystal data

[Cu(ClO_4_)(C_2_H_3_N)(C_15_H_19_N_3_O)]ClO_4_
*M* *_r_* = 560.83
Monoclinic, 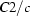

*a* = 18.8394 (16) Å
*b* = 10.6049 (9) Å
*c* = 23.171 (2) Å
β = 102.998 (2)°
*V* = 4510.7 (7) Å^3^
*Z* = 8
Mo *K*α radiation
μ = 1.26 mm^−1^
*T* = 200 K
0.20 × 0.17 × 0.08 mm

#### Data collection

Siemens SMART CCD diffractometer
Absorption correction: multi-scan (*SADABS*; Sheldrick, 1996 ▶) *T*_min_ = 0.777, *T*_max_ = 0.904
16472 measured reflections
5616 independent reflections
3249 reflections with *I* > 2σ(*I*)
*R*_int_ = 0.066

#### Refinement

*R*[*F* ^2^ > 2σ(*F* ^2^)] = 0.061
*wR*(*F* ^2^) = 0.188
*S* = 1.11
5616 reflections
303 parameters
H atoms treated by a mixture of independent and constrained refinement
Δρ_max_ = 1.66 e Å^−3^
Δρ_min_ = −1.16 e Å^−3^

### 

Data collection: *SMART* (Siemens, 1996 ▶); cell refinement: *SAINT* (Siemens, 1996 ▶); data reduction: *SAINT*; program(s) used to solve structure: *SHELXS97* (Sheldrick, 2008 ▶); program(s) used to refine structure: *SHELXL97* (Sheldrick, 2008 ▶); molecular graphics: *ORTEP-3* (Farrugia, 1997 ▶); software used to prepare material for publication: *SHELXL97*.

## Supplementary Material

Crystal structure: contains datablocks global, I. DOI: 10.1107/S1600536810053985/zs2085sup1.cif

Structure factors: contains datablocks I. DOI: 10.1107/S1600536810053985/zs2085Isup2.hkl

Additional supplementary materials:  crystallographic information; 3D view; checkCIF report

## Figures and Tables

**Table 1:** Hydrogen-bond geometry (Å, °)

*D*—H⋯*A*	*D*—H	H⋯*A*	*D*⋯*A*	*D*—H⋯*A*
O1—H1*A*⋯O9	0.74 (5)	2.46 (5)	3.090 (12)	145 (5)

**Fig. 1.**
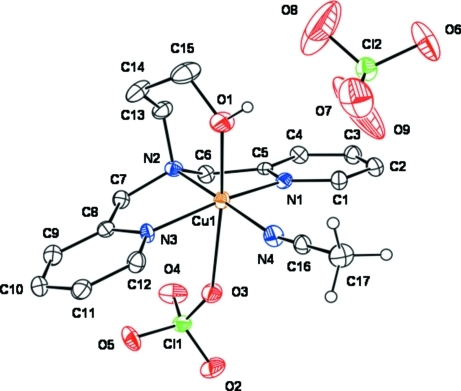
Molecular configuration and atom numbering scheme for the title compound, with 30% probability displacement ellipsoids. Non-H atoms are omitted.

**Fig. 2.**
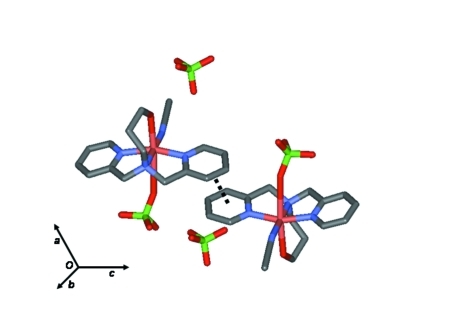
Perspective view of the title compound showing the offset π–π stacking interaction via one of the pyridine groups of the ligand, (indicated as a dashed line).

**Fig. 3.**
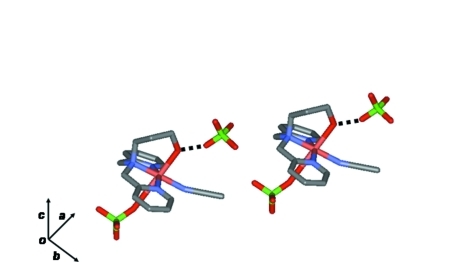
Perspective view of the title compound showing an hydroxyl O—H···O (perchlorate) hydrogen-bonding interaction, indicated as a dashed line.

## supplementary crystallographic information

### Comment

The preparation and characterization of polyamine complexes have allowed the elucidatation of the mechanisms of metalloenzyme reactions (Tshuva & Lippard, 2004). The complexes with bis(2-pyridylmethyl)amine moieties have been widely studied (Bebout *et al.*,1998; Shin *et al.*, 2010) because the tridentate unit has potential in biological applications (van Staveren *et al.*, 2002), examples being the Pd^II^ and Pt^II^ complexes with bis(2-pyridylmethyl)amine or its derivatives, as anticancer agents, e.g. *cis*-platin (Rauterkus *et al.*, 2003). Here, we report the synthesis and crystal structure of a six-coordidine Cu^II^ complex with *N*,*N*-bis(2-pyridylmethyl)-3-aminopropanol (bpapOH), the title compound [Cu(bpapOH)(CH_3_CN)(ClO_4_)] ClO_4_ (I).

In the title compound (Fig. 1) the copper(II) ion is bonded to three N atoms of the tetradentate ligand and one N atom from an acetonitrile solvent molecule in an equatorial plane and two O atoms in axial sites, one from the hydroxyl group of the ligand, the other from a perchlorate ion, resulting in a tetragonally distorted octahedral stereochemistry. The bond lengths around Cu^II^ in the equatorial plane are in the range of 1.986 (4)–2.021 (4) Å while the axial Cu–O distances are 2.232 (4) Å (hydroxy) and 2.868 (4) Å (perchlorate), due to Jahn-Teller distortion. The bond angles about the copper atom lie in the range 84.12 (17)–178.30 (19)°. One of the pyridyl groups of the coordinated bpapOH ligand (N1–C5) is involved in a an offset face-to-face π–π inter-complex stacking interaction (Shetty *et al.*, 1996) (ring centroid separation  Cg1···Cg^i^, 3.718 (2) Å], giving dimers (Fig. 2) [symmetry code: (i) -*x* + 1/2, -*y* + 1/2, -*z* + 1]. The inter-planar separation of these pyridine rings is 3.491 (2) Å and the dihedral angle between the pyridine ring planes is 0.0°. Additionally, an intra-complex O—H···O hydrogen-bonding interaction is found between the hydroxyl group of the ligand and the free perchlorate anion (Table 1) (Fig. 3).

### Experimental

A MeOH solution (5 ml) of Cu(ClO_4_)_2_ . 6H_2_O (72 mg, 0.194 mmol) was added to a MeOH solution (5 ml) of *N*,*N*-bis(2-pyridylmethyl)-3-aminopropanol (bpapOH) (50 mg, 0.194 mmol) (Young *et al.*, 1995). The mixture was stirred for 10 min at room temperature, resulting in a color change to blue-green. Diffusion of diethylether into the mixture gave blue crystals of the title compound after a few days and these were washed with diethyl ether and dried in air (yield: 43 mg, 40%). FTIR (KBr, cm^-1^): *ν*(OH), 3393; *ν*(ClO_4_^-^), 1087, 627; *ν*(C—H), 3070, 2862; *ν*(C—N), 1607.

### Refinement

All C-bound H atoms in the title compound were placed in geometrically idealized positions and constrained to ride on their parent atoms, with C—H distances of 0.95 Å (ring H atoms) and 0.98–0.99 Å (open chain H atoms), and with *U*_iso_(H) values of 1.2 or 1.5*U*_iso_ of the parent C atoms. The hydroxyl H atom was located in a difference Fourier and its position and *U*_iso_ value were allowed to refine freely.

### Crystal data

**Table:**

[Cu(ClO_4_)(C_2_H_3_N)(C_15_H_19_N_3_O)]ClO_4_	*F*(000) = 2296
*M**_r_* = 560.83	*D*_x_ = 1.652 Mg m^−^^3^
Monoclinic, *C*2/*c*	Mo *K*α radiation, λ = 0.71073 Å
Hall symbol: -C 2yc	Cell parameters from 3827 reflections
*a* = 18.8394 (16) Å	θ = 2.2–27.2°
*b* = 10.6049 (9) Å	µ = 1.26 mm^−^^1^
*c* = 23.171 (2) Å	*T* = 200 K
β = 102.998 (2)°	Block, blue
*V* = 4510.7 (7) Å^3^	0.20 × 0.17 × 0.08 mm
*Z* = 8	

### Data collection

**Table:**

Siemens SMART CCD diffractometer	5616 independent reflections
Radiation source: fine-focus sealed tube	3249 reflections with *I* > 2σ(*I*)
graphite	*R*_int_ = 0.066
φ and ω scans	θ_max_ = 28.3°, θ_min_ = 1.8°
Absorption correction: multi-scan (*SADABS*; Sheldrick, 1996)	*h* = −25→25
*T*_min_ = 0.777, *T*_max_ = 0.904	*k* = −14→14
16472 measured reflections	*l* = −26→30

### Refinement

**Table:**

Refinement on *F*^2^	Primary atom site location: structure-invariant direct methods
Least-squares matrix: full	Secondary atom site location: difference Fourier map
*R*[*F*^2^ > 2σ(*F*^2^)] = 0.061	Hydrogen site location: inferred from neighbouring sites
*wR*(*F*^2^) = 0.188	H atoms treated by a mixture of independent and constrained refinement
*S* = 1.11	*w* = 1/[σ^2^(*F*_o_^2^) + (0.0574*P*)^2^ + 23.552*P*] where *P* = (*F*_o_^2^ + 2*F*_c_^2^)/3
5616 reflections	(Δ/σ)_max_ = 0.004
303 parameters	Δρ_max_ = 1.66 e Å^−^^3^
0 restraints	Δρ_min_ = −1.16 e Å^−^^3^

### Special details

**Table:**

Geometry. All e.s.d.'s (except the e.s.d. in the dihedral angle between two l.s. planes)are estimated using the full covariance matrix. The cell e.s.d.'s are takeninto account individually in the estimation of e.s.d.'s in distances, anglesand torsion angles; correlations between e.s.d.'s in cell parameters are onlyused when they are defined by crystal symmetry. An approximate (isotropic)treatment of cell e.s.d.'s is used for estimating e.s.d.'s involving l.s.planes.
Refinement. Refinement of *F*^2^ against ALL reflections. The weighted *R*-factor *wR* and goodness of fit *S* are based on *F*^2^, conventional *R*-factors *R* are based on *F*, with *F* set to zero for negative *F*^2^. The threshold expression of *F*^2^ > σ(*F*^2^) is used only for calculating *R*-factors(gt) *etc*. and is not relevant to the choice of reflections for refinement. *R*-factors based on *F*^2^ are statistically about twice as large as those based on *F*, and *R*- factors based on ALL data will be even larger.

### Fractional atomic coordinates and isotropic or equivalent isotropic displacement parameters (Å^2^)

**Table:**

	*x*	*y*	*z*	*U*_iso_*/*U*_eq_	
Cu1	0.13311 (3)	0.19042 (6)	0.63867 (3)	0.0267 (2)	
Cl1	0.35845 (8)	0.20097 (13)	0.70690 (6)	0.0348 (3)	
Cl2	−0.11763 (8)	0.33890 (14)	0.50981 (7)	0.0397 (4)	
N1	0.1499 (2)	0.1811 (4)	0.55716 (19)	0.0263 (9)	
N2	0.1661 (2)	0.0086 (4)	0.6439 (2)	0.0279 (10)	
N3	0.1492 (2)	0.1799 (4)	0.72643 (18)	0.0266 (9)	
N4	0.1036 (3)	0.3717 (4)	0.6338 (2)	0.0352 (11)	
O1	0.0140 (2)	0.1484 (4)	0.6184 (2)	0.0464 (12)	
H1A	−0.016 (3)	0.176 (5)	0.596 (2)	0.056 (16)*	
O2	0.4077 (3)	0.2983 (5)	0.7002 (3)	0.0691 (15)	
O3	0.2870 (2)	0.2349 (4)	0.67406 (19)	0.0432 (11)	
O4	0.3794 (2)	0.0851 (4)	0.6847 (2)	0.0569 (13)	
O5	0.3560 (3)	0.1857 (5)	0.76703 (18)	0.0567 (13)	
O6	−0.1520 (3)	0.3725 (7)	0.4524 (2)	0.085 (2)	
O7	−0.1319 (4)	0.4221 (6)	0.5530 (3)	0.092 (2)	
O8	−0.1411 (9)	0.2274 (8)	0.5240 (4)	0.247 (8)	
O9	−0.0464 (4)	0.3278 (15)	0.5138 (3)	0.230 (7)	
C1	0.1296 (3)	0.2643 (5)	0.5134 (2)	0.0325 (12)	
H1	0.1038	0.3377	0.5203	0.039*	
C2	0.1449 (3)	0.2472 (6)	0.4586 (3)	0.0367 (13)	
H2	0.1300	0.3076	0.4280	0.044*	
C3	0.1825 (3)	0.1403 (5)	0.4491 (3)	0.0362 (13)	
H3	0.1942	0.1269	0.4117	0.043*	
C4	0.2031 (3)	0.0532 (5)	0.4936 (2)	0.0339 (13)	
H4	0.2278	−0.0218	0.4872	0.041*	
C5	0.1870 (3)	0.0769 (5)	0.5481 (2)	0.0254 (11)	
C6	0.2135 (3)	−0.0071 (5)	0.6006 (2)	0.0314 (12)	
H6A	0.2124	−0.0961	0.5876	0.038*	
H6B	0.2644	0.0147	0.6198	0.038*	
C7	0.2068 (3)	−0.0160 (5)	0.7060 (2)	0.0296 (12)	
H7A	0.2598	−0.0110	0.7080	0.035*	
H7B	0.1958	−0.1024	0.7176	0.035*	
C8	0.1873 (3)	0.0770 (5)	0.7490 (2)	0.0288 (12)	
C9	0.2105 (3)	0.0616 (6)	0.8098 (3)	0.0370 (13)	
H9	0.2383	−0.0101	0.8257	0.044*	
C10	0.1923 (3)	0.1526 (6)	0.8468 (3)	0.0402 (15)	
H10	0.2082	0.1449	0.8886	0.048*	
C11	0.1511 (3)	0.2539 (6)	0.8227 (3)	0.0379 (14)	
H11	0.1364	0.3153	0.8475	0.045*	
C12	0.1315 (3)	0.2653 (5)	0.7627 (3)	0.0334 (13)	
H12	0.1041	0.3370	0.7461	0.040*	
C13	0.1030 (3)	−0.0805 (5)	0.6262 (3)	0.0394 (14)	
H13A	0.0839	−0.0730	0.5829	0.047*	
H13B	0.1215	−0.1676	0.6343	0.047*	
C14	0.0411 (4)	−0.0619 (6)	0.6559 (3)	0.0504 (18)	
H14A	0.0601	−0.0263	0.6959	0.061*	
H14B	0.0189	−0.1448	0.6607	0.061*	
C15	−0.0177 (4)	0.0262 (6)	0.6211 (4)	0.058 (2)	
H15A	−0.0363	−0.0070	0.5806	0.070*	
H15B	−0.0589	0.0319	0.6410	0.070*	
C16	0.0700 (3)	0.4605 (5)	0.6291 (2)	0.0291 (12)	
C17	0.0256 (3)	0.5744 (5)	0.6235 (3)	0.0404 (14)	
H17A	0.0255	0.6155	0.5856	0.061*	
H17B	0.0459	0.6322	0.6561	0.061*	
H17C	−0.0244	0.5521	0.6252	0.061*	

### Atomic displacement parameters (Å^2^)

**Table:**

	*U*^11^	*U*^22^	*U*^33^	*U*^12^	*U*^13^	*U*^23^
Cu1	0.0314 (4)	0.0225 (3)	0.0273 (4)	0.0038 (3)	0.0086 (3)	0.0016 (3)
Cl1	0.0357 (8)	0.0375 (7)	0.0332 (7)	−0.0068 (6)	0.0120 (6)	−0.0047 (6)
Cl2	0.0440 (9)	0.0379 (8)	0.0365 (8)	0.0053 (6)	0.0074 (7)	0.0025 (6)
N1	0.030 (2)	0.020 (2)	0.030 (2)	0.0003 (18)	0.0085 (19)	−0.0011 (18)
N2	0.022 (2)	0.032 (2)	0.030 (2)	−0.0014 (19)	0.0068 (19)	−0.002 (2)
N3	0.036 (2)	0.027 (2)	0.017 (2)	0.0041 (19)	0.0062 (18)	0.0031 (18)
N4	0.041 (3)	0.032 (3)	0.033 (3)	0.008 (2)	0.009 (2)	−0.001 (2)
O1	0.021 (2)	0.044 (3)	0.068 (3)	0.004 (2)	−0.003 (2)	0.011 (2)
O2	0.053 (3)	0.069 (3)	0.082 (4)	−0.030 (3)	0.007 (3)	0.012 (3)
O3	0.031 (2)	0.045 (2)	0.051 (3)	−0.0031 (19)	0.0049 (19)	0.009 (2)
O4	0.044 (3)	0.055 (3)	0.074 (3)	0.006 (2)	0.018 (2)	−0.026 (3)
O5	0.072 (3)	0.077 (3)	0.021 (2)	0.001 (3)	0.010 (2)	0.000 (2)
O6	0.072 (4)	0.147 (6)	0.034 (3)	0.040 (4)	0.005 (3)	0.016 (3)
O7	0.128 (6)	0.089 (4)	0.060 (4)	0.026 (4)	0.025 (4)	−0.015 (3)
O8	0.53 (2)	0.092 (6)	0.131 (9)	−0.135 (10)	0.107 (12)	−0.010 (6)
O9	0.078 (5)	0.55 (2)	0.059 (5)	0.122 (9)	0.014 (4)	0.020 (8)
C1	0.038 (3)	0.031 (3)	0.029 (3)	0.003 (2)	0.007 (2)	0.005 (2)
C2	0.041 (3)	0.042 (3)	0.027 (3)	−0.002 (3)	0.006 (3)	0.003 (3)
C3	0.041 (3)	0.039 (3)	0.029 (3)	0.000 (3)	0.011 (3)	0.000 (3)
C4	0.033 (3)	0.036 (3)	0.033 (3)	0.004 (2)	0.007 (2)	−0.003 (3)
C5	0.022 (3)	0.024 (2)	0.027 (3)	−0.006 (2)	0.000 (2)	0.001 (2)
C6	0.031 (3)	0.030 (3)	0.033 (3)	0.007 (2)	0.007 (2)	0.001 (2)
C7	0.029 (3)	0.029 (3)	0.032 (3)	0.005 (2)	0.007 (2)	0.004 (2)
C8	0.024 (3)	0.033 (3)	0.029 (3)	−0.006 (2)	0.008 (2)	0.002 (2)
C9	0.039 (3)	0.038 (3)	0.035 (3)	0.001 (3)	0.009 (3)	0.005 (3)
C10	0.053 (4)	0.044 (3)	0.024 (3)	−0.011 (3)	0.009 (3)	0.003 (3)
C11	0.044 (4)	0.036 (3)	0.036 (3)	−0.008 (3)	0.013 (3)	0.000 (3)
C12	0.042 (3)	0.031 (3)	0.031 (3)	0.002 (3)	0.016 (3)	0.002 (2)
C13	0.034 (3)	0.028 (3)	0.055 (4)	−0.002 (2)	0.006 (3)	0.007 (3)
C14	0.044 (4)	0.034 (3)	0.077 (5)	−0.007 (3)	0.023 (4)	0.010 (3)
C15	0.032 (4)	0.043 (4)	0.099 (6)	−0.002 (3)	0.013 (4)	0.004 (4)
C16	0.034 (3)	0.030 (3)	0.023 (3)	0.002 (2)	0.007 (2)	0.001 (2)
C17	0.036 (3)	0.032 (3)	0.052 (4)	0.010 (3)	0.010 (3)	−0.001 (3)

### Geometric parameters (Å, °)

**Table:**

Cu1—N1	1.986 (4)	C3—H3	0.9500
Cu1—N3	1.991 (4)	C4—C5	1.386 (7)
Cu1—N4	1.997 (5)	C4—H4	0.9500
Cu1—N2	2.021 (4)	C5—C6	1.500 (7)
Cu1—O1	2.232 (4)	C6—H6A	0.9900
Cu1—O3	2.868 (4)	C6—H6B	0.9900
Cl1—O5	1.413 (4)	C7—C8	1.505 (7)
Cl1—O2	1.420 (5)	C7—H7A	0.9900
Cl1—O4	1.421 (4)	C7—H7B	0.9900
Cl1—O3	1.436 (4)	C8—C9	1.387 (8)
Cl2—O8	1.330 (8)	C9—C10	1.385 (8)
Cl2—O9	1.330 (7)	C9—H9	0.9500
Cl2—O6	1.389 (5)	C10—C11	1.368 (8)
Cl2—O7	1.405 (5)	C10—H10	0.9500
N1—C1	1.333 (7)	C11—C12	1.363 (8)
N1—C5	1.349 (6)	C11—H11	0.9500
N2—C7	1.493 (7)	C12—H12	0.9500
N2—C6	1.495 (7)	C13—C14	1.496 (8)
N2—C13	1.501 (7)	C13—H13A	0.9900
N3—C12	1.328 (7)	C13—H13B	0.9900
N3—C8	1.346 (7)	C14—C15	1.532 (9)
N4—C16	1.126 (7)	C14—H14A	0.9900
O1—C15	1.434 (8)	C14—H14B	0.9900
O1—H1A	0.73 (6)	C15—H15A	0.9900
C1—C2	1.377 (8)	C15—H15B	0.9900
C1—H1	0.9500	C16—C17	1.459 (7)
C2—C3	1.381 (8)	C17—H17A	0.9800
C2—H2	0.9500	C17—H17B	0.9800
C3—C4	1.374 (8)	C17—H17C	0.9800
			
N1—Cu1—N3	161.51 (18)	N2—C6—C5	109.8 (4)
N1—Cu1—N4	95.48 (18)	N2—C6—H6A	109.7
N3—Cu1—N4	95.08 (18)	C5—C6—H6A	109.7
N1—Cu1—N2	84.12 (17)	N2—C6—H6B	109.7
N3—Cu1—N2	84.88 (17)	C5—C6—H6B	109.7
N4—Cu1—N2	178.30 (19)	H6A—C6—H6B	108.2
N1—Cu1—O1	99.08 (19)	N2—C7—C8	112.0 (4)
N3—Cu1—O1	96.80 (19)	N2—C7—H7A	109.2
N4—Cu1—O1	85.79 (19)	C8—C7—H7A	109.2
N2—Cu1—O1	95.91 (17)	N2—C7—H7B	109.2
O5—Cl1—O2	111.0 (3)	C8—C7—H7B	109.2
O5—Cl1—O4	109.3 (3)	H7A—C7—H7B	107.9
O2—Cl1—O4	110.3 (3)	N3—C8—C9	120.6 (5)
O5—Cl1—O3	108.4 (3)	N3—C8—C7	117.5 (5)
O2—Cl1—O3	108.6 (3)	C9—C8—C7	121.8 (5)
O4—Cl1—O3	109.2 (3)	C10—C9—C8	118.8 (6)
O8—Cl2—O9	106.9 (9)	C10—C9—H9	120.6
O8—Cl2—O6	110.8 (6)	C8—C9—H9	120.6
O9—Cl2—O6	109.5 (4)	C11—C10—C9	119.4 (6)
O8—Cl2—O7	104.8 (6)	C11—C10—H10	120.3
O9—Cl2—O7	111.0 (6)	C9—C10—H10	120.3
O6—Cl2—O7	113.5 (4)	C12—C11—C10	119.1 (6)
C1—N1—C5	119.6 (5)	C12—C11—H11	120.5
C1—N1—Cu1	127.6 (4)	C10—C11—H11	120.5
C5—N1—Cu1	112.8 (3)	N3—C12—C11	122.4 (5)
C7—N2—C6	111.8 (4)	N3—C12—H12	118.8
C7—N2—C13	111.0 (4)	C11—C12—H12	118.8
C6—N2—C13	107.5 (4)	C14—C13—N2	116.2 (5)
C7—N2—Cu1	108.0 (3)	C14—C13—H13A	108.2
C6—N2—Cu1	106.7 (3)	N2—C13—H13A	108.2
C13—N2—Cu1	111.8 (3)	C14—C13—H13B	108.2
C12—N3—C8	119.7 (5)	N2—C13—H13B	108.2
C12—N3—Cu1	127.3 (4)	H13A—C13—H13B	107.4
C8—N3—Cu1	112.9 (3)	C13—C14—C15	112.6 (6)
C16—N4—Cu1	162.4 (5)	C13—C14—H14A	109.1
C15—O1—Cu1	125.4 (4)	C15—C14—H14A	109.1
C15—O1—H1A	98 (4)	C13—C14—H14B	109.1
Cu1—O1—H1A	130 (5)	C15—C14—H14B	109.1
N1—C1—C2	122.0 (5)	H14A—C14—H14B	107.8
N1—C1—H1	119.0	O1—C15—C14	108.4 (5)
C2—C1—H1	119.0	O1—C15—H15A	110.0
C1—C2—C3	118.5 (5)	C14—C15—H15A	110.0
C1—C2—H2	120.8	O1—C15—H15B	110.0
C3—C2—H2	120.8	C14—C15—H15B	110.0
C4—C3—C2	120.0 (5)	H15A—C15—H15B	108.4
C4—C3—H3	120.0	N4—C16—C17	179.1 (6)
C2—C3—H3	120.0	C16—C17—H17A	109.5
C3—C4—C5	118.7 (5)	C16—C17—H17B	109.5
C3—C4—H4	120.7	H17A—C17—H17B	109.5
C5—C4—H4	120.7	C16—C17—H17C	109.5
N1—C5—C4	121.1 (5)	H17A—C17—H17C	109.5
N1—C5—C6	116.8 (5)	H17B—C17—H17C	109.5
C4—C5—C6	122.0 (5)		
			
N3—Cu1—N1—C1	−137.9 (6)	N4—Cu1—O1—C15	−170.7 (6)
N4—Cu1—N1—C1	−13.4 (5)	N2—Cu1—O1—C15	9.4 (6)
N2—Cu1—N1—C1	168.3 (5)	Cu1—N1—C1—C2	179.7 (4)
O1—Cu1—N1—C1	73.2 (5)	Cu1—N1—C5—C4	179.2 (4)
N3—Cu1—N1—C5	41.3 (7)	C1—N1—C5—C6	175.0 (5)
N4—Cu1—N1—C5	165.9 (4)	Cu1—N1—C5—C6	−4.3 (6)
N2—Cu1—N1—C5	−12.5 (3)	C3—C4—C5—C6	−174.2 (5)
O1—Cu1—N1—C5	−107.5 (3)	C7—N2—C6—C5	−151.1 (4)
N1—Cu1—N2—C7	145.7 (3)	C13—N2—C6—C5	86.9 (5)
N3—Cu1—N2—C7	−19.4 (3)	Cu1—N2—C6—C5	−33.2 (5)
O1—Cu1—N2—C7	−115.7 (3)	N1—C5—C6—N2	25.8 (6)
N1—Cu1—N2—C6	25.3 (3)	C4—C5—C6—N2	−157.7 (5)
N3—Cu1—N2—C6	−139.8 (3)	C6—N2—C7—C8	139.1 (4)
O1—Cu1—N2—C6	123.9 (3)	C13—N2—C7—C8	−100.9 (5)
N1—Cu1—N2—C13	−91.9 (4)	Cu1—N2—C7—C8	21.9 (5)
N3—Cu1—N2—C13	103.0 (4)	C12—N3—C8—C9	−2.4 (8)
O1—Cu1—N2—C13	6.6 (4)	Cu1—N3—C8—C9	172.8 (4)
N1—Cu1—N3—C12	134.6 (6)	C12—N3—C8—C7	−179.2 (5)
N4—Cu1—N3—C12	10.0 (5)	N2—C7—C8—N3	−12.6 (7)
N2—Cu1—N3—C12	−171.7 (5)	N2—C7—C8—C9	170.7 (5)
O1—Cu1—N3—C12	−76.3 (5)	C7—C8—C9—C10	178.1 (5)
N1—Cu1—N3—C8	−40.2 (7)	C9—C10—C11—C12	−2.7 (9)
N4—Cu1—N3—C8	−164.8 (4)	Cu1—N3—C12—C11	−173.7 (4)
N2—Cu1—N3—C8	13.5 (4)	C7—N2—C13—C14	69.5 (6)
O1—Cu1—N3—C8	108.9 (4)	C6—N2—C13—C14	−168.0 (5)
N1—Cu1—N4—C16	102.7 (16)	Cu1—N2—C13—C14	−51.2 (6)
N3—Cu1—N4—C16	−92.5 (16)	N2—C13—C14—C15	91.7 (7)
N1—Cu1—O1—C15	94.4 (6)	Cu1—O1—C15—C14	15.4 (9)
N3—Cu1—O1—C15	−76.1 (6)	C13—C14—C15—O1	−63.0 (8)

### Hydrogen-bond geometry (Å, °)

**Table:**

*D*—H···*A*	*D*—H	H···*A*	*D*···*A*	*D*—H···*A*
O1—H1A···O9	0.74 (5)	2.46 (5)	3.090 (12)	145 (5)
